# Relation between laxative use and risk of major bleeding in patients with atrial fibrillation and heart failure

**DOI:** 10.1007/s00380-023-02249-6

**Published:** 2023-02-17

**Authors:** Jumpei Yamamoto, Masaya Yamamoto, Hisao Hara, Yukio Hiroi

**Affiliations:** Department of Cardiology, National Center for Global Health and Medicine, 1-21-1 Toyama, Shinjuku, Tokyo, 162-8655 Japan

**Keywords:** Atrial fibrillation, Laxative, Constipation, Heart failure, Major bleeding

## Abstract

Constipation has been reported to increase the risk of cardiovascular mortality. Patients with atrial fibrillation (AF) and heart failure (HF) have more comorbidities and an increased bleeding risk. However, it remains unclear whether constipation is associated with an increased risk of incident bleeding complications in AF with HF. Here, we investigated the association between constipation requiring laxatives and major bleeding in AF and HF. We retrospectively analyzed the medical records of 370 consecutive patients hospitalized for AF and congestive HF. Constipation was defined as regularly taking laxatives or having at least two prescriptions for a ≥ 30-day supply of laxatives. Sixty patients experienced major bleeding events during a median follow-up of 318 days. The most common sites of bleeding were lower gastrointestinal (28%, 17/60), upper gastrointestinal (27%, 16/60), and intracranial (20%, 12/60). There were 33 (55%) patients with constipation in the bleeding group and 107 (35%) in the non-bleeding group (*P* = 0.004). Multivariate Cox regression analysis adjusted for HAS-BLED score, hemoglobin, and direct oral anticoagulant use showed that constipation (hazard ratio [HR] 1.85, 95% confidence interval [CI] 1.11–3.08; *p* = 0.019) was a significant risk factor for major bleeding. We found a significant association between constipation requiring laxatives and major bleeding in patients with AF and HF. These findings indicate the need for constipation prevention in these patients to avoid reliance on invasive defecation management, including laxatives.

**Supplementary Information:**

The online version contains supplementary material available at 10.1007/s00380-023-02249-6.

## Introduction

Atrial fibrillation (AF) is a common comorbidity in patients with heart failure (HF) and increases the risks of stroke and death [1, 2]. Oral anticoagulant (OAC) therapy is recommended in patients with AF to prevent ischemic stroke and systemic embolism [3] but is associated with an increased bleeding risk [4]. Recent systematic reviews and meta-analyses [5, 6] indicate that direct oral anticoagulants (DOACs) are as effective as vitamin K antagonists (VKAs) in the prevention of non-valvular AF -related ischemic stroke. Although DOAC therapy has been shown to be safer and more effective than VKAs and does not require laboratory-guided dose adjustment [7], it does not completely eliminate complications in patients at high risk for bleeding. Therefore, clinicians still need to assess the risk of stroke and bleeding using CHADS_2_ [8], CHA_2_DS_2_-VASc [9], and HAS-BLED [10] scores. However, determining the indication for anticoagulant therapy is difficult in patients with AF and HF, who may have unknown confounders.

Constipation is common in critically ill patients [11]. The reported global prevalence of constipation is 14%, increases with age, and is twice as common in women than in men [12]. The prevalence of constipation in Japanese patients hospitalized for cardiovascular disease was 47%, half of whom have onset of constipation occurring after admission [13]. Several cohort studies have reported an independent association between constipation requiring laxatives and increased risk of cardiovascular mortality [14, 15]. Another report in a Japanese cohort study found that decreased defecation frequency was associated with an increased risk of cardiovascular mortality [16]. Straining at stool involves Valsalva maneuver-like breathing and has been linked to a transient increase in blood pressure [13], which could be associated with an increased risk of major bleeding. Furthermore, constipation is a frequent symptom of advanced cancer [17] and increases the risk of major bleeding in patients with AF [18]. Taken together, constipation seems to be associated with a risk of major bleeding but the evidence is unclear.

It is likely that the risk of major bleeding is particularly high in patients with AF and HF because all of these patients are indicated for anticoagulant therapy due to a CHADS_2_ score of 1 or higher. In this study, we investigated the association between constipation requiring laxatives and major bleeding in these patients.

## Methods

### Patients

We retrospectively reviewed the electronic medical records of consecutive patients with congestive HF who were emergently admitted to the Department of Cardiology in the Center Hospital of the National Center for Global Health and Medicine (Tokyo, Japan) from December 2013 to December 2018. The inclusion criteria were age ≥ 20 years and a diagnosis of AF based on electrocardiographic or echocardiographic findings. We excluded patients with acute coronary syndromes that could be complicated by AF in the acute phase. Information on baseline characteristics, medications, laboratory data, and follow‐up events was collected for the 370 patients who were eligible for the study. All patients were followed up from admission until a major bleeding event or death, or were censored at the date of last contact or on May 31, 2021 and were grouped according to constipation status and the incidence of major bleeding events.

### Data collection

Patient characteristics at baseline included age, sex, body mass index, blood pressure, New York Heart Association functional classification at admission, and coexisting conditions, including history of smoking, stroke, bleeding, coronary artery disease, valvular heart disease, hypertrophic cardiomyopathy, dilated cardiomyopathy, congenital heart disease, peripheral arterial disease, hypertension, dyslipidemia, diabetes, alcohol-related disorders, active malignancy [19], gastrointestinal diseases (ulcerative disease, malignant disease, liver disease, and constipation [14, 15]). CHADS_2_, CHA_2_DS_2_-VASc, and HAS-BLED scores were also calculated based on baseline characteristics. Medications at discharge included calcium channel blockers, diuretics (loop diuretic, mineralocorticoid receptor antagonist, thiazide, or tolvaptan), antiplatelet agents, non-steroidal anti-inflammatory drugs except for antiplatelet agents, OACs (VKAs or DOACs), angiotensin-converting enzyme inhibitors or angiotensin-receptor blockers, beta-blockers, antiarrhythmics, statins, and proton pump inhibitors. Laboratory data collected on admission included estimated glomerular filtration rate (eGFR), hemoglobin (Hb), brain natriuretic peptide (BNP), and prothrombin time-international normalized ratio (PT-INR). Echocardiographic parameters included left ventricular ejection fraction obtained by the Teichholz method and left atrial diameter from the long-axis view. Follow-up events, including major bleeding events, were assessed.

### Definition

The clinical diagnosis of congestive HF was based on the Framingham criteria [20] and was established by cardiologists on admission. Major bleeding was defined according to the International Society on Thrombosis and Hemostasis criteria [21], which include fatal bleeding, symptomatic bleeding in a critical area or organ, bleeding leading to transfusion of ≥ 2 units of blood, or a ≥ 2 g/dL decrease in hemoglobin. Active malignancy was defined as cancer diagnosed within the previous 12 months or ongoing cancer treatment (surgery, radiotherapy, chemotherapy, or immunotherapy) [19]. The most common definition of constipation, as described in the World Gastroenterology Organization Global Guidelines, is taking laxatives [22]. Here, as in previous studies [14, 15], constipation was defined as regularly taking laxatives at the start of follow-up or receiving at least two 30-day prescriptions for laxatives during follow-up. Given that laxatives vary by region and country, and some drugs are not used in Japan, laxatives were defined as the seven categories of oral medications in the Japanese Society of Gastroenterology guidelines on chronic constipation: probiotics, bulk-forming laxatives, osmotic laxatives (saline laxatives, disaccharide laxatives, lubricant laxatives), stimulants laxatives (anthraquinones, diphenyls), epithelial function-altering agents (chloride channel activators, guanylate cyclase-C agonists), gastroprokinetic agents (5-HT_4_ receptor stimulants), and Chinese herbal medicine [23]. Valvular heart disease was defined as patients with moderate or severe valvular disease according to the clinical practice guidelines [24] and those with a history of surgery for valvular disease. Time in therapeutic range (TTR) was calculated for patients on a VKA who had at least three available PT-INR values during follow-up. In this study, the target PT-INR range was 2.0–3.0 for patients aged < 70 years and 1.6–2.6 for those aged ≥ 70 years in accordance with the Japanese guidelines [25]. TTR was calculated using the Rosendaal method [26], excluding PT-INR values obtained during hospitalization and periods of anticoagulation.

### Statistical analysis

Continuous variables are shown as the mean ± standard deviation or the median and 25th/75th percentiles (interquartile range). Categorical variables are shown as the frequency and percentage. We compared baseline variables between groups using Student’s *t*-test or the Mann–Whitney *U* test if they were continuous and Fisher’s exact test if they were categorical. Univariate and multivariate logistic regression analyses were performed to identify independent risk factors for constipation. Factors identified as significant in univariate analysis were included in multivariate analysis. Odds ratios (ORs) and 95% confidence intervals (CIs) were calculated. The estimated major bleeding-free survival rate was compared between patients with and without constipation using the Kaplan–Meier method and log-rank test. Univariate and multivariate Cox regression analyses were performed to identify independent risk factors for major bleeding. Factors identified as significant in univariate analysis were included in multivariate analysis. Hazard ratios (HRs) and 95% CIs were calculated. Sensitivity analyses were performed on the results. We performed two subgroup analyses. Patients with a higher bleeding risk than thrombotic risk may not have been on anticoagulant therapy at the clinician's discretion. Thus, to reduce this selection bias, only patients receiving OAC therapy were included in one of the subgroup analyses. In addition, because laxative use affects the gastrointestinal system, the second subgroup analysis was performed for only gastrointestinal major bleeding. Patients with missing data were excluded from the analysis. A *p* value less than 0.05 (two-tailed) was considered statistically significant. All statistical analyses were performed using R version 4.1.2 (The R Foundation for Statistical Computing, Vienna, Austria).

## Results

**Fig. 1:**
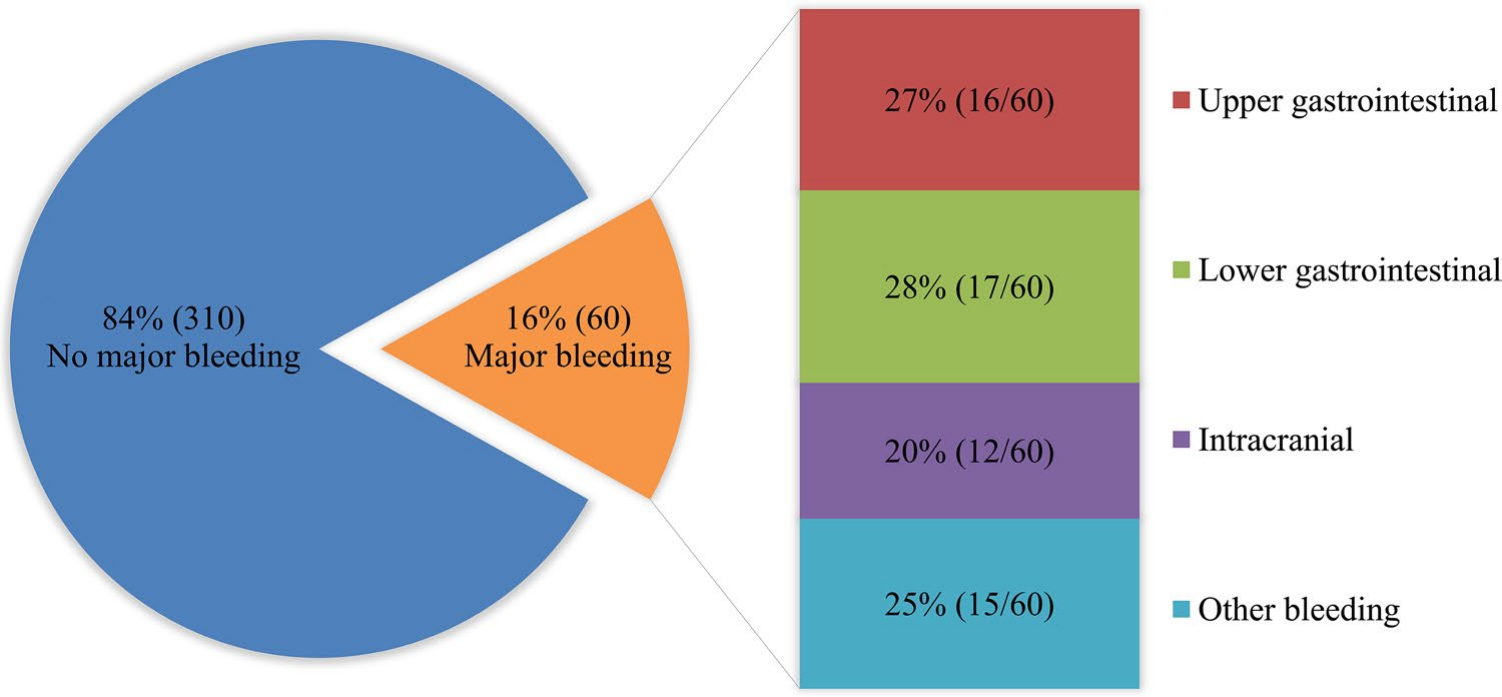
Incidence of major bleeding and bleeding sites. Bleeding sites are shown in patients with atrial fibrillation and heart failure who developed major bleeding. Data are shown as the percentage (number) of patients by bleeding site

**Fig. 2:**
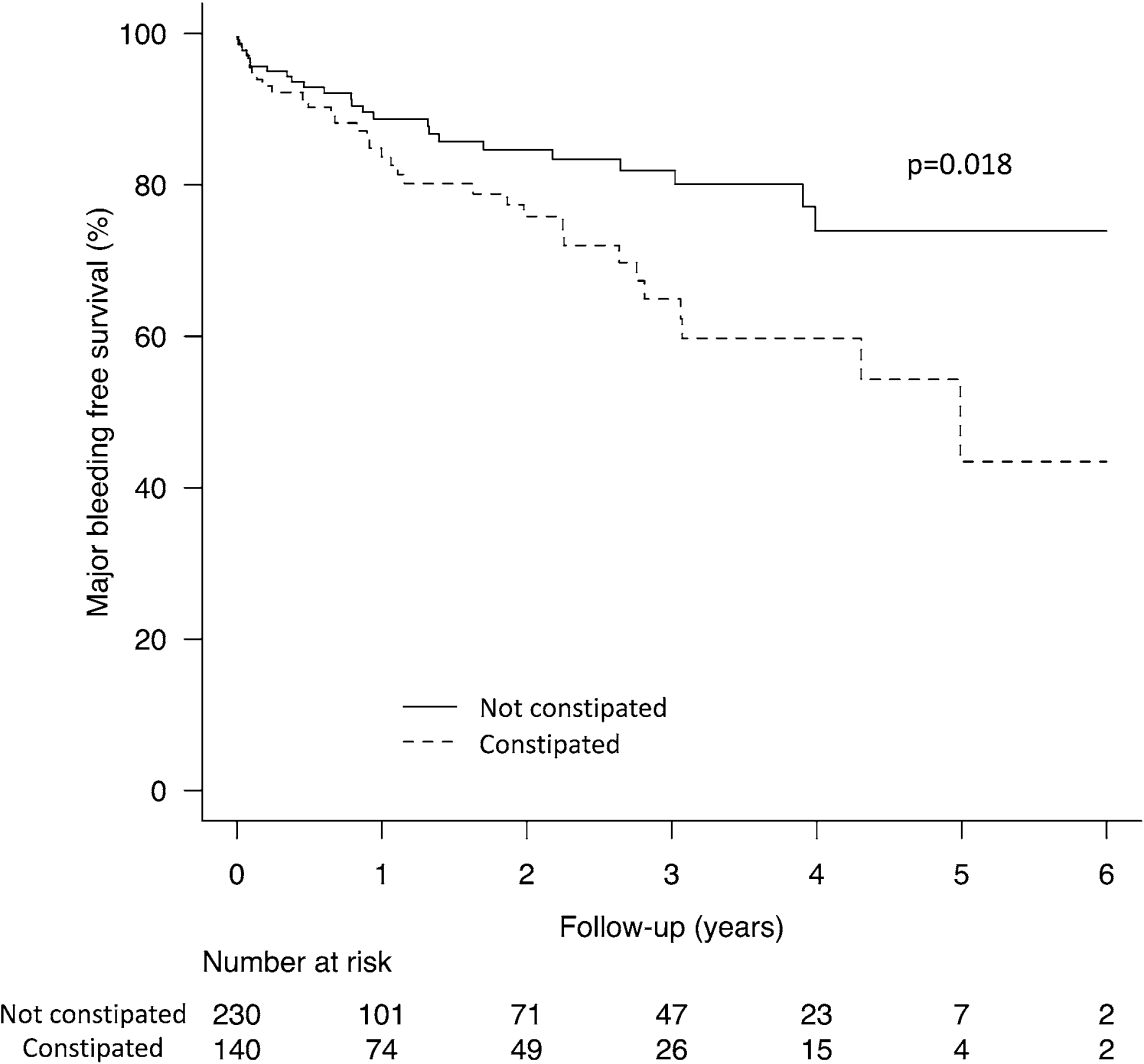
Kaplan–Meier curves for major bleeding free survival. The Kaplan–Meier curves for major bleeding free survival is shown for patients with atrial fibrillation and heart failure according to constipation status. Dash line, constipation group; solid line, non-constipation group

During a median follow-up of 318 days (interquartile range, 56–905), 16% of patients (60/370) experienced major bleeding events. Mean patient age was 79 ± 11 years, 49% (181/370) were women, and 38% (140/370) had constipation. Table 1 shows the baseline characteristics and medications according to constipation status. Patients in the constipation group had significantly higher age, lower Hb, and higher prevalence of active malignancy, but no significant risk factors in multivariate logistic regression analysis. Figure 1 shows the most common sites of bleeding, which were lower gastrointestinal (28%, 17/60), upper gastrointestinal (27%, 16/60), and intracranial (20%, 12/60). There were 4 fatal bleeding events. Table 2 shows the baseline characteristics and medications according to whether or not major bleeding events occurred. There was no significant difference in the use of proton pump inhibitors between the groups. Patients in the bleeding group had significantly higher mean baseline CHADS_2_ and HAS-BLED scores, a significantly higher prevalence of constipation and use of antiplatelet agents, VKAs, and OACs + antiplatelet agents, and significantly lower eGFR, Hb, and use of DOACs. Figure 2 shows the Kaplan–Meier curves for major bleeding-free survival according to constipation status. The 3-year survival rate was 65% (95% CI 52–75) in patients with constipation and 82% (95% CI 74–88; *p* = 0.018) in those without constipation. Multivariate Cox regression analysis was performed. Table 3 shows the independent risk factors for major bleeding with HRs and 95% CIs. Model 1 was adjusted for the HAS-BLED score, Hb, and use of DOACs. The CHADS_2_ and CHA_2_DS_2_-VASc scores as well as the factors included in the HAS-BLED score were excluded because they were significantly correlated. In the multivariate analysis, constipation (HR 1.85, 95% CI 1.11–3.08; *p* = 0.019) was a significant risk factor for major bleeding. Model 2 was adjusted for age, Hb, eGFR, and the use of DOACs and antiplatelet agents. The CHADS_2_, CHA_2_DS_2_-VASc, and HAS-BLED scores were excluded because they were significantly correlated. In multivariate analysis, constipation (HR 1.88, 95% CI 1.12–3.16; *p* = 0.017) was a significant risk factor for major bleeding. Sensitivity analyses were performed by changing DOAC in the multivariate model to VKA, OAC, or OAC + antiplatelets. The results were consistent with constipation being associated with a risk of major bleeding (Supplementary Table S1–3; Additional file 1). In the subgroup analysis performed for only patients who received OAC therapy (Supplementary Table S4–5; Additional file 1), hypertension was more common in the major bleeding group, but the multivariate analysis results were similar to those of the main analysis. In the subgroup analysis performed for only patients with gastrointestinal major bleeding (Supplementary Table S6–7; Additional file 1), alcohol-related disorders, low BNP, and poor TTR were more common in the major bleeding group. Also, there was a stronger association between constipation and major bleeding in the multivariate analysis adjusted for HAS-BLED score and BNP than in the main analysis (HR 2.60, 95% CI 1.27–5.33; *p* = 0.009).

**Table 1:** Patient characteristics according to constipation status

	Constipation		*p* value
	No (*n* = 230)	Yes (*n* = 140)	
Age, years	78.3 ± 12.5	81.0 ± 9.4	**0.022**
Female sex, *n*	105 (45.7)	76 (54.3)	0.11
Body mass index, kg/m^2^	21.7 ± 4.5	21.4 ± 5.4	0.55
Blood pressure			
Systolic, mmHg	131 ± 27	128 ± 26	0.30
Diastolic, mmHg	83 ± 22	79 ± 20	0.065
LVEF, %	48 ± 16	49 ± 16	0.48
LAD, mm	49 ± 9	50 ± 10	0.47
BNP	618 (402–1074)	612 (355–987)	0.51
NYHA functional class			0.21
II, *n*	26 (11.3)	16 (11.4)	
III, *n*	77 (33.5)	59 (42.1)	
IV, *n*	127 (55.2)	65 (46.4)	
Hb, mg/dL	12.4 ± 2.7	11.8 ± 2.0	**0.037**
eGFR, mL/min/1.73 m^2^	47 ± 22	45 ± 19	0.35
CHADS_2_ score	3.0 ± 1.2	3.1 ± 1.2	0.35
CHA_2_DS_2_-VASc score	4.6 ± 1.5	4.9 ± 1.4	0.15
HAS-BLED score	2.5 ± 1.4	2.6 ± 1.2	0.30
History of smoking, *n*	99 (43.0)	58 (41.4)	0.83
Comorbidity			
Coronary artery disease, *n*	58 (25.2)	28 (20.0)	0.26
Valvular heart disease, *n*	66 (28.7)	47 (33.6)	0.35
Hypertrophic cardiomyopathy, *n*	2 (0.9)	6 (4.3)	0.057
Dilated cardiomyopathy, *n*	2 (0.9)	4 (2.9)	0.21
Congenital heart disease, *n*	1 (0.4)	2 (1.4)	0.56
Peripheral arterial disease, *n*	5 (2.2)	4 (2.9)	0.74
Hypertension, *n*	173 (75.2)	106 (75.7)	1.00
Dyslipidemia, *n*	68 (29.6)	47 (33.6)	0.42
Diabetes, *n*	69 (30.0)	45 (32.1)	0.73
Alcohol-related disorders, n	0 (0.0)	3 (2.1)	0.053
Active malignancy, *n*	3 (1.3)	7 (5.0)	**0.046**
History of stroke, *n*	36 (15.7)	21 (15.0)	1.00
History of major bleeding, *n*	12 (5.2)	13 (9.3)	0.14
Gastrointestinal diseases			
Ulcerative disease	18 (7.8)	14 (10.0)	0.57
Malignant disease	19 (8.3)	16 (11.4)	0.36
Liver disease, *n*	5 (2.2)	7 (5.0)	0.22
Medication at discharge			
OAC, *n*	188 (81.7)	117 (83.6)	0.68
VKA, *n*	94 (40.9)	60 (42.9)	0.75
TTR, %	56 ± 30	58 ± 27	0.66
DOAC, *n*	94 (40.9)	57 (40.7)	1.00
Antiplatelets, *n*	64 (27.8)	37 (26.4)	0.81
OAC + antiplatelets, *n*	49 (21.3)	28 (20.0)	0.79
NSAIDs, *n*	4 (1.7)	2 (1.4)	1.00
PPIs, *n*	129 (56.1)	93 (66.4)	0.050
Diuretics, *n*	208 (90.4)	130 (92.9)	0.45
Loop diuretics, *n*	204 (88.7)	125 (89.3)	1.00
MRA, *n*	112 (48.7)	58 (41.4)	0.20
Thiazide, *n*	20 (8.7)	15 (10.7)	0.58
Tolvaptan, *n*	30 (13.0)	17 (12.1)	0.87
Beta-blockers, *n*	166 (72.2)	98 (70.0)	0.72
ACEI/ARB, *n*	97 (42.2)	54 (38.6)	0.51
Calcium channel blockers, *n*	87 (37.8)	65 (46.4)	0.13
Antiarrhythmics, *n*	20 (8.7)	17 (12.1)	0.29
Statins, *n*	62 (27.0)	43 (30.7)	0.48

Data are presented as the number (percentage), mean ± standard deviation, or median (interquartile range). Bold indicates significance at *p* < 0.05

*ACEI* angiotensin-converting enzyme inhibitor, *ARB* angiotensin receptor blocker, *BNP* brain natriuretic peptide, *DOAC* direct oral anticoagulant, *Hb* hemoglobin, *eGFR* estimated glomerular filtration rate, *LAD* left atrial diameter, *LVEF* left ventricular ejection fraction, *MRA* mineralocorticoid receptor antagonist, *NSAIDs* non-steroidal anti-inflammatory drugs, *NYHA* New York Heart Association, *OAC* oral anticoagulant, *TTR* time in therapeutic range, *VKA* vitamin K antagonist

**Table 2:** Patient characteristics according to major bleeding status

	Major bleeding		*p* value
	No (*n* = 310)	Yes (*n* = 60)	
Age, years	79.5 ± 11.8	78.9 ± 10.0	0.72
Female sex, *n*	154 (49.7)	27 (45.0)	0.57
Body mass index, kg/m^2^	21.5 ± 4.4	22.3 ± 7.0	0.23
Blood pressure			
Systolic, mmHg	130 ± 26	131 ± 29	0.87
Diastolic, mmHg	82 ± 22	78 ± 19	0.18
LVEF, %	48 ± 16	52 ± 15	0.082
LAD, mm	49 ± 9	52 ± 10	0.055
BNP	617 (384–1070)	633 (287–890)	0.35
NYHA functional class			0.12
II, *n*	32 (10.3)	10 (16.7)	
III, *n*	120 (38.7)	16 (26.7)	
IV, *n*	158 (51.0)	34 (56.7)	
Hb, mg/dL	12.3 ± 2.5	11.3 ± 2.5	**0.006**
eGFR, mL/min/1.73 m^2^	47 ± 21	40 ± 20	**0.020**
CHADS_2_ score	3.0 ± 1.1	3.4 ± 1.3	**0.041**
CHA_2_DS_2_-VASc score	4.7 ± 1.5	5.0 ± 1.5	0.067
HAS-BLED score	2.4 ± 1.3	3.2 ± 1.2	** < 0.001**
History of smoking, *n*	132 (42.6)	25 (41.7)	1.00
Comorbidity			
Coronary artery disease, *n*	68 (21.9)	18 (30.0)	0.18
Valvular heart disease, *n*	97 (31.3)	16 (26.7)	0.54
Hypertrophic cardiomyopathy, *n*	7 (2.3)	1 (1.7)	1.00
Dilated cardiomyopathy, *n*	4 (1.3)	2 (3.3)	0.25
Congenital heart disease, *n*	3 (1.0)	0 (0.0)	1.00
Peripheral arterial disease, *n*	9 (2.9)	0 (0.0)	0.37
Hypertension, *n*	228 (73.5)	51 (85.0)	0.071
Dyslipidemia, *n*	93 (30.0)	22 (36.7)	0.36
Diabetes, *n*	92 (29.7)	22 (36.7)	0.29
Alcohol-related disorders, *n*	1 (0.3)	2 (3.3)	0.070
Active malignancy, *n*	10 (3.2)	0 (0.0)	0.38
History of stroke, *n*	44 (14.2)	13 (21.7)	0.17
History of major bleeding, *n*	20 (6.5)	5 (8.3)	0.58
Gastrointestinal diseases			
Ulcerative disease	24 (7.7)	8 (13.3)	0.21
Malignant disease	25 (8.1)	10 (16.7)	0.051
Liver disease, *n*	8 (2.6)	4 (6.7)	0.11
Constipation, *n*	107 (34.5)	33 (55.0)	**0.004**
Medication at discharge			
OAC, *n*	259 (83.5)	46 (76.7)	0.20
VKA, *n*	122 (39.4)	32 (53.3)	**0.047**
TTR, %	58 ± 28	52 ± 31	0.30
DOAC, *n*	137 (44.2)	14 (23.3)	**0.003**
Antiplatelets, *n*	75 (24.2)	26 (43.3)	**0.004**
OAC + antiplatelets, *n*	58 (18.7)	19 (31.7)	**0.036**
NSAIDs, *n*	6 (1.9)	0 (0.0)	0.60
PPIs, *n*	181 (58.4)	41 (68.3)	0.20
Diuretics, *n*	281 (90.6)	57 (95.0)	0.45
Loop diuretics, *n*	274 (88.4)	55 (91.7)	0.65
MRA, *n*	147 (47.4)	23 (38.3)	0.21
Thiazide, *n*	28 (9.0)	7 (11.7)	0.48
Tolvaptan, *n*	37 (11.9)	10 (16.7)	0.30
Beta-blockers, *n*	221 (71.3)	43 (71.7)	1.00
ACEI/ARB, *n*	121 (39.0)	30 (50.0)	0.12
Calcium channel blockers, *n*	127 (41.0)	25 (41.7)	1.00
Antiarrhythmics, *n*	31 (10.0)	6 (10.0)	1.00
Statins, *n*	83 (26.8)	22 (36.7)	0.12

Data are presented as the number (percentage), mean ± standard deviation, or median (interquartile range). Bold indicates significance at *P* < 0.05

*ACEI* angiotensin-converting enzyme inhibitor, *ARB* angiotensin receptor blocker, *BNP* brain natriuretic peptide, *DOAC* direct oral anticoagulant, *eGFR* estimated glomerular filtration rate, *Hb* hemoglobin, *LAD* left atrial diameter, *LVEF* left ventricular ejection fraction, *MRA* mineralocorticoid receptor antagonist, *NSAIDs* non-steroidal anti-inflammatory drugs, *NYHA* New York Heart Association, *OAC* oral anticoagulant, *TTR* time in therapeutic range, *VKA* vitamin K antagonist

**Table 3:** Multivariate analyses of risk factors for major bleeding events

	Model 1			Model 2		
Risk factor	HR	95% CI	*p* value	HR	95% CI	*p* value
HAS-BLED score	1.35	1.12–1.64	**0.002**			
Age				0.99	0.96–1.02	0.36
Hb	0.82	0.73–0.93	**0.001**	0.81	0.71–0.92	**0.001**
eGFR				0.99	0.98–1.01	0.35
Constipation	1.85	1.11–3.08	**0.019**	1.88	1.12–3.16	**0.017**
DOAC	0.48	0.25–0.91	**0.024**	0.44	0.23–0.83	**0.011**
Antiplatelets				1.84	1.08–3.11	**0.024**

Bold indicates significance at *P* < 0.05

*CI* confidence interval, *DOAC* direct oral anticoagulant, *eGFR* estimated glomerular filtration rate, *Hb* hemoglobin, *HR* hazard ratio

## Discussion

In this study, we investigated the independent risk factors for major bleeding in patients with AF and HF. The high prevalence of constipation of 38% in this study was similar to that in a previous study of patients with cardiovascular disease [13], suggesting that fluid intake restriction and the use of diuretics and calcium channel blockers in the management of HF may predispose to constipation [27]. In this study, the incidence of major bleeding events was 16%, which is more than twice that in a prospective cohort study of patients with AF in Japan [28]. All patients in our study had HF and were indicated for OAC therapy, which may have led to the inclusion of patients at high risk of bleeding. However, in the multivariate analysis adjusted for use of DOACs, constipation was identified as a significant risk factor for major bleeding, and consistent results were obtained in sensitivity analyses where DOACs were replaced with VKAs, OACs, or OAC + antiplatelet agents. Considering the possibility that patients at high risk of bleeding did not receive OACs at the discretion of clinicians, a subgroup analysis was performed for only patients who received OACs, but the results were consistent. These results suggest that constipation may contribute to the risk of major bleeding in patients indicated for OAC therapy, regardless of the type of OAC or whether they take them or not. In addition, the association was even stronger in the subgroup analysis for only gastrointestinal major bleeding. To our knowledge, this is the first report of an independent association between constipation and major bleeding in patients with AF and HF.

Two prospective community-based cohort studies focused on constipation as a risk factor for atherosclerotic cardiovascular disease [14, 29]. Both studies reported that patients with constipation had higher risks of all-cause mortality, coronary heart disease, and ischemic stroke than their counterparts without constipation. Similarly, another cohort study reported that constipation was associated with increased risks of HF, AF, and hemorrhagic stroke [15]. Indeed, the patients in our study were much older and had more comorbidities than those in these common population cohorts. Therefore, the overall prevalence of constipation and the incidence of bleeding events observed in our study tended to be higher than in the cohort studies. Furthermore, the increased risk of cardiovascular disease has been reported to be highest in the first year after onset of constipation [15]. This indicates that there is a greater likelihood of complications in patients who develop constipation due to fluid restriction, medications that cause constipation, or impaired exercise tolerance after hospitalization for HF. Thus, in contrast with previous studies, we investigated the association of constipation with overall major bleeding in patients with AF and HF, who are known to be a group at higher risk of bleeding.

A simple explanation for our results is that straining at stool with constipation could have been associated with a risk of major bleeding. Straining at stool, which involves Valsalva maneuver-like breathing, could have caused an increase in intrathoracic pressure, leading to an increase in systolic blood pressure [13]. It has been reported that systolic blood pressure can increase by 30–70 mmHg in the elderly during defecation and the increase persists for 1 h [13]. Repeated daily fluctuations in blood pressure resembling a morning surge could injure vascular endothelial cells, and then damage to large and small blood vessels could contribute to bleeding risk by promoting atherosclerosis and causing organ damage [30]. Because this mechanism is a chronic process, the risk of bleeding might have remained even in patients with constipation that was well-controlled by laxatives. Other mechanisms could involve constipation triggering an inflammatory process that in turn accelerates development of bleeding events. Overgrowth of the intestinal microbiota due to constipation can cause release of cytokines by activating macrophages, which may induce atherosclerosis, elevated blood pressure, and cardiovascular events [31–33]. The richness of gut microbiota species is reduced in patients with AF and may be associated with insulin resistance, dyslipidemia, and inflammation [34]. A recent study reported that persistent systemic inflammation was associated with increased bleeding risk in patients with AF [35]; thus, chronic inflammation caused by constipation may increase the risk of bleeding, especially in patients with AF. Moreover, other studies have found that constipation may also be a risk factor for development of chronic kidney disease (CKD) [36, 37] and for all-cause mortality in hemodialysis patients [38]. One possible cause of this is that constipation may increase the concentration of toxic substances in the blood and urine [39]. It has also been reported that patients with end-stage CKD and AF could have an increased risk of bleeding due to difficulties in anticoagulant management [40, 41].

This study also identified a low baseline hemoglobin level to be a significant risk factor for major bleeding in patients with HF and AF. The mechanism of the association between low hemoglobin and increased bleeding risk is not known, but low baseline hemoglobin level might reflect occult gastrointestinal bleeding. It has also been reported that a low hematocrit level decreases the platelet activity, which was thought to be due to margination of fewer platelets to near vessel walls as a result of the low red blood cell count [42]. Our results suggest that it is necessary to examine the cause of the anemia and determine the indication for anticoagulation therapy. It is also known that combination antiplatelet and anticoagulation therapy increases the risk of bleeding [43] but no significant increase in risk was observed in the multivariate analysis of this study. In the subgroup analysis performed in only patients who received OAC therapy, the risk of bleeding was significantly lower with DOACs, which may have affected the risk of bleeding with combination therapy. This finding suggests that combination therapy with a VKA should be avoided when possible. Although constipation might be merely one of the frequent symptoms of advanced cancer [17], a recent study found a positive association between constipation and some types of cancer [44]. Another cohort study reported that advanced cancer was associated with a risk of major bleeding in patients with AF [18]. However, our study found no major bleeding in patients with active malignancy, so another mechanism may have been involved in the increase in bleeding events. Although the mechanism of the association between constipation and a high incidence of bleeding remains unclear, our findings indicate a need for constipation control and adjustment of constipation-producing medications in patients at high risk of bleeding.

This study had some limitations. First, it had a small sample size and a retrospective design and was conducted at a single center. Second, information on the actual stool pattern and symptoms of constipation was not available. Because the clinical definition of constipation was not symptom-based [45], patients with constipation who had no laxative prescription records might have been misclassified as not having constipation. Third, surveillance and detection bias cannot be excluded. However, major bleeding is characterized by a severe clinical course, which makes this research less prone to surveillance bias. Finally, we cannot exclude the possibility of unmeasured confounders, such as type of AF. There has been a recent report of an independent association between sustained AF and an increased risk of bleeding in patients with HF [46]. In view of the small number of patients in our study who used antiarrhythmic agents, most patients likely had persistent AF, which could have contributed to the risk of bleeding.

## Conclusions

In this study, we found that constipation requiring laxatives could be associated with major bleeding in patients with AF and HF. Our findings suggest that these patients are more likely to develop constipation, which can lead to bleeding complications. Further studies in a prospective cohort are needed to confirm the association and to elucidate the pathophysiology of bleeding complications caused by constipation. Controlled trials are also needed to determine whether constipation prevention without reliance on invasive defecation control, including laxatives, reduces bleeding complications in these patients.

## Supplementary Information

Below is the link to the electronic supplementary material.

Supplementary file1 (DOCX 34 KB)

## Data Availability

The data underlying this article cannot be shared publicly without compromising the privacy of the study participants but are available from the corresponding author on reasonable request.

## Abbreviations

ACEI: Angiotensin-converting enzyme inhibitor
AF: Atrial fibrillation
ARB: Angiotensin receptor blocker
BNP: Brain natriuretic peptide
CI: Confidence interval
CKD: Chronic kidney disease
DOAC: Direct oral anticoagulant
eGFR: Estimated glomerular filtration rate
Hb: Hemoglobin
HF: Heart failure
HR: Hazard ratio
MRA: Mineralocorticoid receptor antagonist
N/A: Not applicable
NSAIDs: Non-steroidal anti-inflammatory drugs
OAC: Oral anticoagulant
OR: Odds ratios
TTR: Time in therapeutic range
VKA: Vitamin K antagonist
