# Supplementary material for: Relation between laxative use and risk of major bleeding in patients with atrial fibrillation and heart failure
Source: Heart Vessels. 2023 Feb 17;38(7):938–48. doi: 10.1007/s00380-023-02249-6 (PMC10209255; doi:10.1007/s00380-023-02249-6)
Supplement: Supplementary file 1 — Supplementary file1 (DOCX 34 KB) [file 380_2023_2249_MOESM1_ESM.docx]

Table S1. Multivariate analysis of risk factors for major bleeding events with DOAC replaced with VKA in the model

|  | Model 1 | | | Model 2 | | |
| --- | --- | --- | --- | --- | --- | --- |
| Risk factor | HR | 95% CI | *P*-value | HR | 95% CI | *P*-value |
| HAS-BLED score | 1.46 | 1.21–1.76 | **<0.001** |  |  |  |
| Age |  |  |  | 0.99 | 0.96-1.01 | 0.31 |
| Hb | 0.81 | 0.71-0.91 | **<0.001** | 0.79 | 0.70-0.91 | **<0.001** |
| eGFR |  |  |  | 0.99 | 0.98-1.00 | 0.11 |
| Constipation | 1.81 | 1.08-3.03 | **0.024** | 1.82 | 1.08–3.04 | **0.023** |
| VKA | 1.31 | 0.78–2.20 | 0.30 | 1.31 | 0.78–2.20 | 0.30 |
| Antiplatelets |  |  |  | 2.01 | 1.19–3.38 | **0.009** |

Bold indicates significance at *P* < 0.05. CI, confidence interval; eGFR, estimated glomerular filtration rate; Hb, hemoglobin; HR, hazard ratio; VKA, vitamin K antagonist

Table S2. Multivariate analysis of risk factors for major bleeding events with DOAC replaced with OAC in the model

|  | Model 1 | | | Model 2 | | |
| --- | --- | --- | --- | --- | --- | --- |
| Risk factor | HR | 95% CI | *P*-value | HR | 95% CI | *P*-value |
| HAS-BLED score | 1.39 | 1.15–1.69 | **<0.001** |  |  |  |
| Age |  |  |  | 0.98 | 0.96-1.01 | 0.22 |
| Hb | 0.83 | 0.73-0.94 | **0.004** | 0.81 | 0.71-0.93 | **0.002** |
| eGFR |  |  |  | 0.99 | 0.98-1.01 | 0.27 |
| Constipation | 1.74 | 1.04–2.90 | **0.034** | 1.75 | 1.05–2.93 | **0.032** |
| OAC | 0.58 | 0.29–1.19 | 0.14 | 0.49 | 0.24–0.98 | **0.043** |
| Antiplatelets |  |  |  | 1.86 | 1.09–3.19 | **0.023** |

Bold indicates significance at *P* < 0.05. CI, confidence interval; eGFR, estimated glomerular filtration rate; Hb, hemoglobin; HR, hazard ratio; OAC, oral anticoagulant

Table S3. Multivariate analysis of risk factors for major bleeding events with DOAC replaced with OAC + antiplatelets in the model

|  | Model 1 | | | Model 2 | | |
| --- | --- | --- | --- | --- | --- | --- |
| Risk factor | HR | 95% CI | *P*-value | HR | 95% CI | *P*-value |
| HAS-BLED score | 1.45 | 1.20–1.76 | **<0.001** |  |  |  |
| Age |  |  |  | 0.98 | 0.96-1.01 | 0.25 |
| Hb | 0.81 | 0.71-0.92 | **<0.001** | 0.80 | 0.70-0.91 | **<0.001** |
| eGFR |  |  |  | 0.99 | 0.97-0.99 | **0.034** |
| Constipation | 1.78 | 1.07–2.98 | **0.027** | 1.73 | 1.03–2.89 | **0.037** |
| OAC + antiplatelets | 1.17 | 0.65–2.08 | 0.60 | 1.73 | 0.99–3.00 | 0.052 |

Bold indicates significance at *P* < 0.05. CI, confidence interval; eGFR, estimated glomerular filtration rate; Hb, hemoglobin; HR, hazard ratio; OAC, oral anticoagulant

Table S4. Patient characteristics according to major bleeding status in only patients who received OAC therapy

|  | Major bleeding | |  |
| --- | --- | --- | --- |
|  | No (n=259) | Yes (n=46) | *P-*value |
| Age, years | 78.5 ± 11.9 | 77.2 ± 9.6 | 0.48 |
| Female sex, n | 127 (49.0) | 18 (39.1) | 0.26 |
| Body mass index, kg/m^2^ | 21.7 ± 4.2 | 23.0 ± 7.5 | 0.10 |
| Blood pressure |  |  |  |
| Systolic, mmHg | 129 ± 26 | 131 ± 31 | 0.56 |
| Diastolic, mmHg | 83 ± 21 | 78 ± 18 | 0.17 |
| LVEF, % | 48 ± 16 | 52 ± 14 | 0.11 |
| LAD, mm | 49 ± 9 | 52 ± 10 | 0.14 |
| BNP | 573 (367-982) | 610 (235-852) | 0.43 |
| NYHA functional class |  |  | 0.065 |
| Ⅱ, n | 29 (11.2) | 9 (19.6) |  |
| Ⅲ, n | 110 (42.5) | 12 (26.1) |  |
| Ⅳ, n | 120 (46.3) | 25 54.3) |  |
| Hb, mg/dL | 12.5 ± 2.3 | 11.7 ± 2.5 | 0.055 |
| eGFR, mL/min/1.73 m^2^ | 49 ± 20 | 44 ± 18 | 0.14 |
| CHADS_2_ score | 2.9 ± 1.1 | 3.4 ± 1.2 | **0.008** |
| CHA₂DS₂-VASc score | 4.5 ± 1.4 | 5.0 ± 1.5 | **0.029** |
| HAS-BLED score | 2.3 ± 1.2 | 3.1 ± 1.2 | **<0.001** |
| History of smoking, n | 110 (42.5) | 20 (43.5) | 1.00 |
| Comorbidity |  |  |  |
| Coronary artery disease, n | 52 (20.1) | 15 (32.6) | 0.080 |
| Valvular heart disease, n | 86 (33.2) | 9 (19.6) | 0.083 |
| Hypertrophic cardiomyopathy, n | 6 (2.3) | 1 (2.2) | 1.00 |
| Dilated cardiomyopathy, n | 4 (1.5) | 1 (2.2) | 0.56 |
| Congenital heart disease, n | 3 (1.2) | 0 (0.0) | 1.00 |
| Peripheral arterial disease, n | 6 (2.3) | 0 (0.0) | 1.00 |
| Hypertension, n | 187 (72.2) | 40 (87.0) | **0.043** |
| Dyslipidemia, n | 80 (30.9) | 19 (41.3) | 0.17 |
| Diabetes, n | 72 (27.8) | 18 (39.1) | 0.16 |
| Alcohol-related disorders, n | 1 (0.4) | 2 (4.3) | 0.060 |
| Active malignancy, n | 8 (3.1) | 0 (0.0) | 0.61 |
| History of stroke, n | 30 (11.6) | 10 (21.7) | 0.093 |
| History of major bleeding, n | 14 (5.4) | 3 (6.5) | 0.73 |
| Gastrointestinal diseases |  |  |  |
| Ulcerative disease | 19 (7.3) | 5 (10.9) | 0.38 |
| Malignant disease | 25 (9.7) | 9 (19.6) | 0.071 |
| Liver disease, n | 6 (2.3) | 3 (6.5) | 0.14 |
| Constipation, n | 91 (35.1) | 26 (56.5) | **0.008** |
| Medication at discharge |  |  |  |
| OAC, n | 259 (100) | 46 (100) | N/A |
| VKA, n | 122 (47.1) | 32 (69.6) | **0.006** |
| TTR, % | 58 ± 28 | 52 ± 31 | 0.30 |
| DOAC, n | 137 (52.9) | 14 (30.4) | **0.006** |
| Antiplatelets, n | 58 (22.4) | 19 (41.3) | **0.010** |
| NSAIDs, n | 6 (2.3) | 0 (0.0) | 0.60 |
| PPIs, n | 154 (59.5) | 29 (63.0) | 0.75 |
| Diuretics, n | 244 (94.2) | 43 (93.5) | 0.74 |
| Loop diuretics, n | 238 (91.9) | 42 (91.3) | 0.78 |
| MRA, n | 132 (51.0) | 18 (39.1) | 0.15 |
| Thiazide, n | 24 (9.3) | 5 (10.9) | 0.78 |
| Tolvaptan, n | 35 (13.5) | 6 (13.0) | 1.00 |
| Beta-blockers, n | 194 (74.9) | 34 (73.9) | 0.86 |
| ACEI/ARB, n | 108 (41.7) | 26 (56.5) | 0.076 |
| Calcium channel blockers, n | 109 (42.1) | 19 (41.3) | 1.00 |
| Antiarrhythmics, n | 27 (10.4) | 5 (10.9) | 1.00 |
| Statins, n | 75 (29.0) | 20 (43.5) | 0.058 |

Data are presented as the number (percentage), mean ± standard deviation, or median (interquartile range). Bold indicates significance at *P* < 0.05. ACEI, angiotensin-converting enzyme inhibitor; ARB, angiotensin receptor blocker; BNP, brain natriuretic peptide; DOAC, direct oral anticoagulant; eGFR, estimated glomerular filtration rate; Hb, hemoglobin; LAD, left atrial diameter; LVEF, left ventricular ejection fraction; MRA, mineralocorticoid receptor antagonist; N/A, not applicable; NSAIDs, non-steroidal anti-inflammatory drugs; NYHA, New York Heart Association; OAC, oral anticoagulant; TTR, time in therapeutic range; VKA, vitamin K antagonist;

Table S5. Multivariate analyses of risk factors for major bleeding events in only patients who received OAC therapy

|  | Model 1 | | | Model 2 | | |
| --- | --- | --- | --- | --- | --- | --- |
| Risk factor | HR | 95% CI | *P*-value | HR | 95% CI | *P*-value |
| HAS-BLED score | 1.55 | 1.24–1.94 | **<0.001** |  |  |  |
| Hypertension |  |  |  | 2.25 | 0.92–5.53 | 0.076 |
| Constipation | 2.10 | 1.17–3.78 | **0.013** | 2.25 | 1.25–4.07 | **0.007** |
| DOAC | 0.59 | 0.30–1.15 | 0.12 | 0.43 | 0.23–0.81 | **0.009** |
| Antiplatelets |  |  |  | 1.74 | 0.94–3.23 | 0.077 |

Bold indicates significance at *P* < 0.05. CI, confidence interval; eGFR, estimated glomerular filtration rate; DOAC, direct oral anticoagulant; HR, hazard ratio; OAC, oral anticoagulant

Table S6. Patient characteristics according to major gastrointestinal bleeding status

|  | Major gastrointestinal bleeding | |  |
| --- | --- | --- | --- |
|  | No (n=337) | Yes (n=33) | *P-*value |
| Age, years | 79.3 ± 11.7 | 80.2 ± 9.9 | 0.66 |
| Female sex, n | 168 (49.9) | 13 (39.4) | 0.28 |
| Body mass index, kg/m^2^ | 21.6 ± 4.9 | 21.8 ± 4.9 | 0.81 |
| Blood pressure |  |  |  |
| Systolic, mmHg | 130 ± 26 | 131 ± 33 | 0.93 |
| Diastolic, mmHg | 82 ± 21 | 75 ± 19 | 0.095 |
| LVEF, % | 48 ± 16 | 53 ± 13 | 0.12 |
| LAD, mm | 49 ± 9 | 52 ± 10 | 0.12 |
| BNP | 640 (392-1072) | 524 (192-742) | **0.030** |
| NYHA functional class |  |  | 0.11 |
| Ⅱ, n | 40 (11.9) | 2 (6.1) |  |
| Ⅲ, n | 128 (38.0) | 8 (24.2) |  |
| Ⅳ, n | 169 (50.1) | 23 (69.7) |  |
| Hb, mg/dL | 12.2 ± 2.5 | 11.4 ± 2.6 | 0.071 |
| eGFR, mL/min/1.73 m^2^ | 46 ± 21 | 40 ± 20 | 0.092 |
| CHADS_2_ score | 3.1 ± 1.2 | 3.2 ± 1.2 | 0.37 |
| CHA₂DS₂-VASc score | 4.7 ± 1.5 | 4.9 ± 1.3 | 0.50 |
| HAS-BLED score | 2.5 ± 1.3 | 3.1 ± 1.1 | **0.011** |
| History of smoking, n | 140 (41.5) | 17 (51.5) | 0.28 |
| Comorbidity |  |  |  |
| Coronary artery disease, n | 76 (22.6) | 10 (30.3) | 0.39 |
| Valvular heart disease, n | 105 (31.2) | 8 (24.2) | 0.55 |
| Hypertrophic cardiomyopathy, n | 8 (2.4) | 0 (0.0) | 1.00 |
| Dilated cardiomyopathy, n | 4 (1.2) | 2 (6.1) | 0.092 |
| Congenital heart disease, n | 3 (0.9) | 0 (0.0) | 1.00 |
| Peripheral arterial disease, n | 9 (2.7) | 0 (0.0) | 1.00 |
| Hypertension, n | 251 (74.5) | 28 (84.8) | 0.21 |
| Dyslipidemia, n | 105 (31.2) | 10 (30.3) | 1.00 |
| Diabetes, n | 103 (30.6) | 11 (33.3) | 0.84 |
| Alcohol-related disorders, n | 1 (0.3) | 2 (6.1) | **0.022** |
| Active malignancy, n | 10 (3.0) | 0 (0.0) | 0.61 |
| History of stroke, n | 52 (15.4) | 5 (15.2) | 1.00 |
| History of major bleeding, n | 22 (6.5) | 3 (9.1) | 0.46 |
| Gastrointestinal diseases |  |  |  |
| Ulcerative disease | 26 (7.7) | 6 (18.2) | 0.052 |
| Malignant disease | 33 (9.8) | 2 (6.1) | 0.76 |
| Liver disease, n | 9 (2.7) | 3 (9.1) | 0.082 |
| Constipation, n | 119 (35.3) | 21 (63.6) | **0.002** |
| Medication at discharge |  |  |  |
| OAC, n | 281 (83.4) | 24 (72.7) | 0.15 |
| VKA, n | 141 (41.8) | 13 (39.4) | 0.86 |
| TTR, % | 59 ± 28 | 37 ± 27 | **0.008** |
| DOAC, n | 140 (41.5) | 11 (33.3) | 0.46 |
| Antiplatelets, n | 87 (25.8) | 14 (42.4) | 0.063 |
| OAC + antiplatelets, *n* | 67 (19.9) | 10 (30.3) | 0.18 |
| NSAIDs, n | 6 (1.8) | 0 (0.0) | 1.00 |
| PPIs, n | 197 (58.5) | 25 (75.8) | 0.063 |
| Diuretics, n | 307 (91.1) | 31 (93.9) | 0.75 |
| Loop diuretics, n | 299 (88.7) | 30 (90.9) | 1.00 |
| MRA, n | 158 (46.9) | 12 (36.4) | 0.28 |
| Thiazide, n | 31 (9.2) | 4 (12.1) | 0.54 |
| Tolvaptan, n | 41 (12.2) | 6 (18.2) | 0.29 |
| Beta-blockers, n | 240 (71.2) | 24 (72.7) | 1.00 |
| ACEI/ARB, n | 136 (40.4) | 15 (45.5) | 0.58 |
| Calcium channel blockers, n | 136 (40.4) | 16 (48.5) | 0.46 |
| Antiarrhythmics, n | 33 (9.8) | 4 (12.1) | 0.76 |
| Statins, n | 93 (27.6) | 12 (36.4) | 0.31 |

Data are presented as the number (percentage), mean ± standard deviation, or median (interquartile range). Bold indicates significance at *P* < 0.05. ACEI, angiotensin-converting enzyme inhibitor; ARB, angiotensin receptor blocker; BNP, brain natriuretic peptide; DOAC, direct oral anticoagulant; eGFR, estimated glomerular filtration rate; Hb, hemoglobin; LAD, left atrial diameter; LVEF, left ventricular ejection fraction; MRA, mineralocorticoid receptor antagonist; N/A, not applicable; NSAIDs, non-steroidal anti-inflammatory drugs; NYHA, New York Heart Association; OAC, oral anticoagulant; TTR, time in therapeutic range; VKA, vitamin K antagonist

Table S7. Multivariate analyses of risk factors for major gastrointestinal bleeding events

|  | Model 1 | | |
| --- | --- | --- | --- |
| Risk factor | HR | 95% CI | *P*-value |
| HAS-BLED score | 1.53 | 1.18–1.98 | **0.001** |
| BNP | 1.00 | 0.99-1.00 | 0.49 |
| Constipation | 2.60 | 1.27–5.33 | **0.009** |

Bold indicates significance at *P* < 0.05. BNP, brain natriuretic peptide; CI, confidence interval; HR, hazard ratio
